# Recurrence of common bile duct stones after endoscopic clearance and its predictors: A systematic review

**DOI:** 10.1002/deo2.294

**Published:** 2023-10-08

**Authors:** Marko Kozyk, Suprabhat Giri, Sidharth Harindranath, Manan Trivedi, Kateryna Strubchevska, Rakesh Kumar Barik, Sridhar Sundaram

**Affiliations:** ^1^ Department of Internal Medicine Corewell Health William Beaumont University Hospital Royal Oak Michigan USA; ^2^ Department of Gastroenterology & Hepatology Kalinga Institute of Medical Sciences Bhubaneswar India; ^3^ Department of Gastroenterology Seth GS Medical College and KEM Hospital Mumbai India; ^4^ Department of General Surgery KB Bhabha Hospital Mumbai India; ^5^ Department of Gastroenterology Indian Institute of Gastroenterology and Hepatology Cuttack India; ^6^ Department of Digestive Diseases and Clinical Nutrition Tata Memorial Hospital Mumbai India

**Keywords:** bile duct stone, cholecystectomy, ERCP, gallstone disease, meta‐analysis

## Abstract

**Background:**

The primary therapeutic strategy for the management of bile duct stones (BDS) is endoscopic retrograde cholangiopancreatography. However, there may be a recurrence of BDS on follow‐up. Multiple risk factors have been studied for the prediction of BDS recurrence. We aimed to analyze the incidence of symptomatic BDS recurrence, systematically review the risk factors, and analyze the most important risk factors among those.

**Methods:**

A comprehensive search of three databases was conducted from inception to November 2022 for studies reporting the recurrence of BDS recurrence after endoscopic retrograde cholangiopancreatography with clearance, along with an analysis of risk factors.

**Results:**

A total of 37 studies with 12,952 patients were included in the final analysis. The pooled event rate for the recurrence of BDS stones was 12.6% (95% confidence interval: 11.2–13.9). The most important risk factor was a bile duct diameter ≥15 mm, which had a significant association with recurrence in twelve studies. Other risk factors with significant association with recurrence in three or more studies were the reduced angulation of the bile duct, the presence of periampullary diverticulum, type I periampullary diverticulum, in‐situ gallbladder with stones, cholecystectomy, multiple stones in the bile duct, use of mechanical lithotripsy, and bile duct stent placement.

**Conclusion:**

Around one out of seven patients have BDS recurrence after the initial endoscopic retrograde cholangiopancreatography. Bile duct size and anatomy are the most important predictors of recurrence. The assessment of risk factors associated with recurrence may help keep a close follow‐up in high‐risk patients.

## INTRODUCTION

Endoscopic retrograde cholangiopancreatography (ERCP) is the modality of choice for the management of common bile duct (CBD) stones in view of minimal morbidity, shorter operative time, fewer complications, and better prognosis than bile duct surgery. Post‐procedure complications are divided into early (within 3 months after the procedure) and late (more than 3 months after the procedure). With an ever‐greater number of ERCPs performed, the most commonly cited long‐term complication in these patients is the recurrence of CBD stones which causes decreased patient satisfaction and increased resource use. The rate of recurrence has been reported to range from 4% to 30% across multiple studies.^1^, ^2^, ^3^, ^4^ CBD stones detected 6 months or more after ERCP are generally considered recurrent as opposed to retained.

The most common factors associated with recurrence in literature are multiple or large CBD stones, intrahepatic stones, pneumobilia, dilated or sharp angles of the CBD, gallstones in the in‐situ gallbladder, delayed biliary emptying, periampullary diverticulum, duodenobiliary reflux, papillary or biliary stricture, systemic disease (e.g., hemolytic anemia), and so on. Several studies have reported the incidence and risk factors for recurrent CBD stones, but the data is disparate. A study by Keizman et al. demonstrated that symptomatic recurrence of CBD stones was found to be significantly more common in elderly patients than in young.^5^ Kim et al. demonstrated that sustained dilatation of the bile duct, even after the complete removal of stones and the location of the papilla on the inner rim or deep within the diverticulum, were independent risk factors for recurrent CBD stones.^6^ Also, the clearance rate with ERCP and endoscopic sphincterotomy or endoscopic papillary balloon dilatation is not impressive as a single, one‐time procedure. In addition, in patients with CBD stones and gallstones, stone migration from the gallbladder to the CBD before and during cholecystectomy also contributes to recurrent stones. A small subset of patients suffer from multiple recurrences with a short recurrence‐free interval. Data on factors predicting recurrence in these patients is sparse. The present meta‐analysis aims to estimate the incidence of and evaluate the risk factors for recurrent CBD stones by performing a systematic review of the available literature.

## METHODS

The current meta‐analysis was conducted as per the Meta‐analysis Of Observational Studies in Epidemiology^7^ and the updated Preferred Reporting Items for Systematic Reviews and Meta‐Analyses (PRISMA) guidelines.^8^

### Database search

Electronic databases of Embase, MEDLINE, and Scopus were searched for titles and abstracts from inception to November 2022 for all relevant studies using the keywords: (ERCP OR Sphincterotomy OR Papillotomy) AND (Choledocholithiasis OR “Bile duct stone” OR “Bile duct calculus”) AND (Recurrence OR Recurrent). Screening of the title and abstract of studies retrieved using the search strategy was done by two independent reviewers. Studies that potentially met the inclusion criteria were extracted. Two researchers independently assessed the full texts before including them. The bibliography of the included studies was also searched for any relevant studies. In case of any disagreement, it was resolved by a third reviewer.

### Study inclusion

Studies included in this analysis were prospective cohort and retrospective case‐control studies fulfilling the following criteria: (a) *Study population* – Patients with CBD stone; (b) *Intervention* – ERCP with CBD clearance; (c) *Outcomes* – recurrence of CBD stone and predictors. Conference abstracts, case reports, case series, studies on pediatric patients, review articles, correspondences, editorials, and studies in languages other than English were excluded.

### Data extraction and quality assessment

Data was collected in a structured data extraction form by two independent reviewers. The form contained the following parameters of each study: title, first author, year of publication, country, number of patients, age and gender, inclusion criteria, outcome measures, and duration of follow‐up. Two independent reviewers assessed the quality of the included studies using the Newcastle‐Ottawa scale.^9^ A third independent individual was consulted to determine the best score based on any discrepancy in the study quality assessment.

### Data analysis

The pooled proportions were computed using a random‐effects inverse‐variance model with a DerSimonian‐Laird estimate of tau^2^.^10^ The heterogeneity was assessed by I^2^ and *p*‐value of heterogeneity. A *p* < 0.10 was taken as statistically significant while I^2^ values of < 25%, 25%–50%, and > 50% were considered as low, moderate, and significant heterogeneity, respectively. The assessment of publication bias was done by evaluation of funnel plot asymmetry and quantified using Egger's test. The meta‐analysis was performed using the Stata 17.0 software package (Stata Corp LP) and RevMan software (version 5.4.1; Cochrane Collaboration). Risk factors that were significantly associated with bile duct stones (BDS) recurrence on multivariate analysis in three or more studies were subsequently discussed.

## RESULTS

### Study characteristics and quality assessment

**FIGURE 1:**
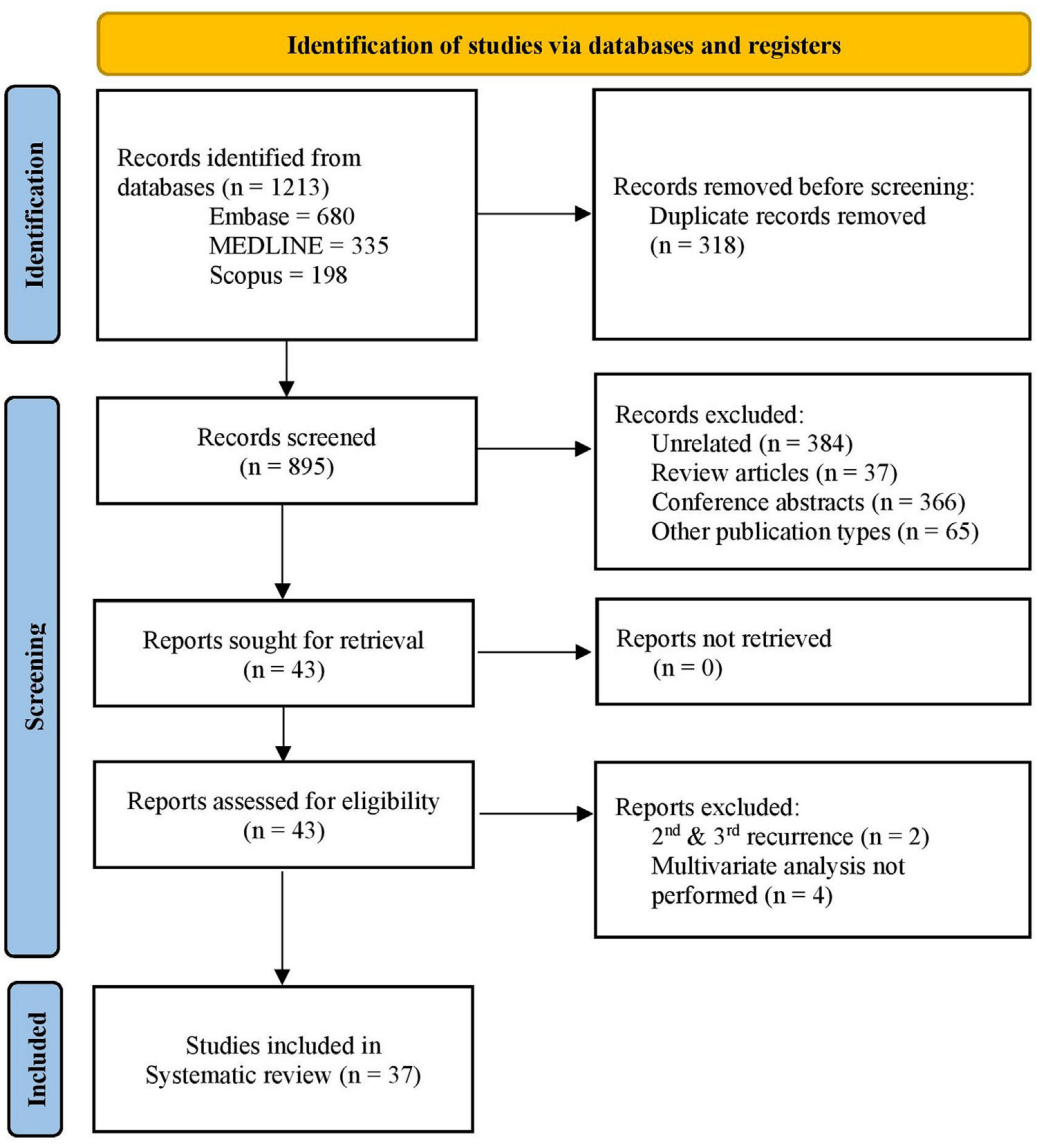
Preferred Reporting Items for Systematic Reviews and Meta‐Analyses (PRISMA) flowchart for study identification, selection, and inclusion process.

A total of 1213 records were identified with the abovementioned search strategy. Figure 1 shows the PRISMA diagram for the study selection and inclusion process. A total of 37 studies with 12,952 patients were included in the final analysis.^1^, ^2^, ^3^, ^4^, ^5^, ^6^, ^11^, ^12^, ^13^, ^14^, ^15^, ^16^, ^17^, ^18^, ^19^, ^20^, ^21^, ^22^, ^23^, ^24^, ^25^, ^26^, ^27^, ^28^, ^29^, ^30^, ^31^, ^32^, ^33^, ^34^, ^35^, ^36^, ^37^, ^38^, ^39^, ^40^, ^41^ Table 1 shows the baseline characteristics of the studies included in the present meta‐analysis, along with the study quality assessment. Among the included studies, only 9 were prospective.^1^, ^3^, ^4^, ^6^, ^11^, ^12^, ^14^, ^20^, ^40^ The majority were from Asia, with only 5 studies from non‐Asian countries.^1^, ^4^, ^20^, ^23^, ^26^ Prior cholecystectomy varied from 7.5% to 100%, while the prevalence of periampullary diverticulum (PAD) varied from 9.7% to 51.6%. Except for three studies,^27^, ^33^, ^40^ the rest were of medium to high quality.

**TABLE 1:** Baseline characteristics of the included studies.

Author, year	Country	Study design	Patient population	No. of patient	Age, in years	Male/ Female	Cholecystectomy	PAD	Follow up duration	Recurrence	Quality
Pereira‐Lima, 1998^1^	Germany	Prospective	EST for BDS	223	72 (38–97)	74/149	127 (56.9%)	57 (25.5%)	6.2 years	16/201 (8.0%)	Fair
Sugiyama, 2002^2^	Japan	Retrospective	EST for BDS with age ≤ 60 years	135	‐	61/74	103 (76.3%)	27 (20%)	14.5 years (6.5–22.3)	12 (8.9%)	Good
Ando, 2003^3^	Japan	Prospective	EST for BDS	983	67	500/483	289 (29.4%)	343 (34.9%)	7.5 years	111 (11.3%)	Fair
Hoem, 2006^4^	Norway	Prospective	BDS extraction	101	‐	‐	‐	‐	7 years	15 (14.8%)	Fair
Keizman, 2006^5^	Israel	Retrospective	EST for BDS	228	63.6 ± 18.3	85/143	‐	60 (26%)	33 months (12–95)	38 (17%)	Good
Tsujino, 2007^11^	Japan	Prospective	EPBD for BDS	1000	69.1 ± 14.6	572/428	132 (13.2%)	385 (38.5%)	4.4 ± 2.8 years	74/837 (8.8%)	Fair
Baek, 2009^12^	Korea	Prospective	EST for BDS	114	63.1 ± 14.5	61/53	23 (20.2%)	53 (46.5%)	12.9 ± 8.8 months	22 (19.3%)	Fair
Ohashi, 2009^13^	Japan	Retrospective	EPBD for BDS	182	69.6 ± 3.7	93/89	34 (18.7%)	89 (48.9%)	9.9 years (7.0 – 12.1)	13 (7.1%)	Fair
Yasuda, 2010^14^	Japan	Prospective	EST vs. EPBD for BDS	282	‐	167/115	42 (14.9%)	123 (43.6%)	6.7 years	36 (12.8%)	Good
Kim, 2011^15^	Korea	Retrospective	EPLBD for BDS	50	67.4 ± 14.4	22/28	17 (34%)	20 (40%)	10.8 ± 4.5 m	12 (24%)	Fair
Kim, 2013^6^	Korea	Prospective	BDS clearance in acute cholangitis	187	66.4 ± 14.1	89/88	41 (21.9%)	74 (39.5%)	‐	16 (8.5%)	Good
Kim, 2013^16^	Korea	Retrospective	EST or EPBD for BDS	222	‐	112/110	39 (17.6%)	123 (55.4%)	25 (6‐48) months	14 (6.3%)	Fair
Chang, 2014^17^	Korea	Retrospective	BDS extraction	481	‐	‐	‐	‐	63.6 months	34 (7.1%)	Fair
Zhang, 2015^a^, ^18^	China	Retrospective	BDS extraction	R: 32, C: 32	R: 56 ± 14, C: 56 ± 14	R: 23/9, C: 23/9	R: 24 (75%), C: 21 (64.3%)	R: 15 (46.9%), C: 9 (28.1%)	R: 6.6 ± 1.5 yrs., C: 7.2 ± 1.8 yrs.	‐	Good
Park, 2016^19^	Korea	Retrospective	EPBD for BDS	128	68.8 ± 13.2	58/70	83 (64.8%)	66 (51.6%)	1398 (136–5455) days	14/107 (13.1%)	Fair
Paspatis, 2016^20^	Greece	Prospective	EPLBD for BDS	106	74.3	46/60	8 (7.5%)	18 (17%)	30.5 ± 5.5 m	8 (7.5%)	Good
Song, 2016^21^	Korea	Retrospective	BDS extraction	317	65.6 ± 12.1	144/173	105 (33.1%)	81 (25.6%)	25.4 ± 22.0 m	64 (20.2%)	Good
Kato, 2017^22^	Japan	Retrospective	BDS extraction in naïve papilla	384	70.8	200/184	‐	136 (35.4%)	1098 days	34 (8.9%)	Fair
Konstantakis, 2017^23^	Greece	Retrospective	EST for BDS	495	‐	‐	‐	‐	35.3 ± 16.9	67 (13.5%)	Good
Li, 2018^24^	China	Retrospective	EST for BDS	345	63 (15–92)	131/214	242 (70.14%)	106 (30.7%)	54 months	57 (16.52%)	Fair
Maruta, 2018^25^	Japan	Retrospective	EST or EPLBD for BDS	240	81 (76–86)	116/124	57 (23.7%)	121 (50.4%)	4.2 years	28 (11.7%)	Good
Nzenza, 2018^26^	Australia	Retrospective	EST for BDS	573	67	‐	‐	92 (16%)	3.3 years (0.5–15 years)	51 (8.9%)	Fair
Yoo, 2018^27^	Korea	Retrospective	EST for BDS followed by cholecystectomy	622	‐	340/282	622 (100%)	244 (39.2%)	66 months (1–252)	115 (18.5%)	Poor
Deng, 2019^a^,^28^	China	Retrospective	BDS extraction	R: 477, C: 477	‐	216/261	‐	‐	‐	‐	Good
Kawaji, 2019^29^	Japan	Retrospective	BDS extraction	976	69.3 ± 13.7	592/384	400 (41%)	373 (38.2%)	5.1 ± 4.4 years	121 (12.4%)	Good
Zhou, 2019^30^	China	Retrospective	EST for BDS	168	57.1 ± 14.8	73/95	18 (10.7%)	‐	6.3 (5.4–7.3) years	18/88 (20.5%)	Good
Lujian, 2020^31^	China	Retrospective		262	63.1 ± 14.1	114/148	101 (38.5%)	79 (30.1%)	20.51 ± 9.65 m	51 (19.5%)	Fair
Murabayashi, 2020^32^	Japan	Retrospective	EPLBD for BDS	93	78.8 ± 8.7	51/47	40 (40.8%)	48 (49.0%)	33.7 ± 16.6 months	16 (17.2%)	Good
Choi, 2021^33^	Korea	Retrospective	BDS extraction	483	62.7 ± 16.2	274/209	336 (69.6%)	47 (9.7%)	3.5 months	54 (11.2%)	Poor
Choi, 2021^34^	Korea	Retrospective	BDS extraction	362	64.8 ± 16.8	188/174	285 (78.7%)	174 (48.1%)	32.3 ± 8.1 months	60 (16.6%)	Fair
Takimoto, 2021^35^	Japan	Retrospective	EST for BDS	243	70.5 ± 11.9	136/107	88 (36.2%)	105 (43.2%)	953.5 ± 776.4 days	33 (13.6%)	Good
Akay, 2022^36^	Turkey	Retrospective	BDS extraction	614	‐	267/347	174 (28.3%)	110 (17.9%)	3.3 ± 2.1 years	85 (13.8%)	Fair
Guan, 2022^37^	China	Retrospective	BDS extraction	218	57.8 ± 17.9	108/110	71 (32.6%)	68 (31.2%)	3 years	40 (18.3%)	Good
Ji, 2022^38^	China	Retrospective	BDS extraction	502	65.2 ± 15.6	287/215	‐	243 (48.4%)	19 months	43	Fair
Li, 2022^39^	China	Retrospective	EPLBD with or without EST	225	70.0 ± 14.2	104/121	119 (52.9%)	111 (49.3%)	38 (27–101) months	29 (12.9%)	Good
Sagami, 2022^40^	Japan	Prospective	BDS extraction and stenting	56	70.7 ± 12.9	36/20	45/56 (80.4%)	‐	112 days	16 (28.5%)	Poor
Sbeit, 2022^41^	Israel	Retrospective	BDS extraction	457	65.3 ± 19.5	215/221	117 (25.6%)	93 (20.3%)	‐	42 (9.2%)	Fair

^a^ Case‐control studies.

Abbreviations: BDS, bile duct stone; EPBD, endoscopic papillary balloon dilatation; EPLBD, endoscopic papillary large balloon dilatation; EST, endoscopic sphincterotomy; PAD, periampullary diverticulum; R/C, recurrence group/ control group.

### Recurrence of BDS after initial clearance

**FIGURE 2:**
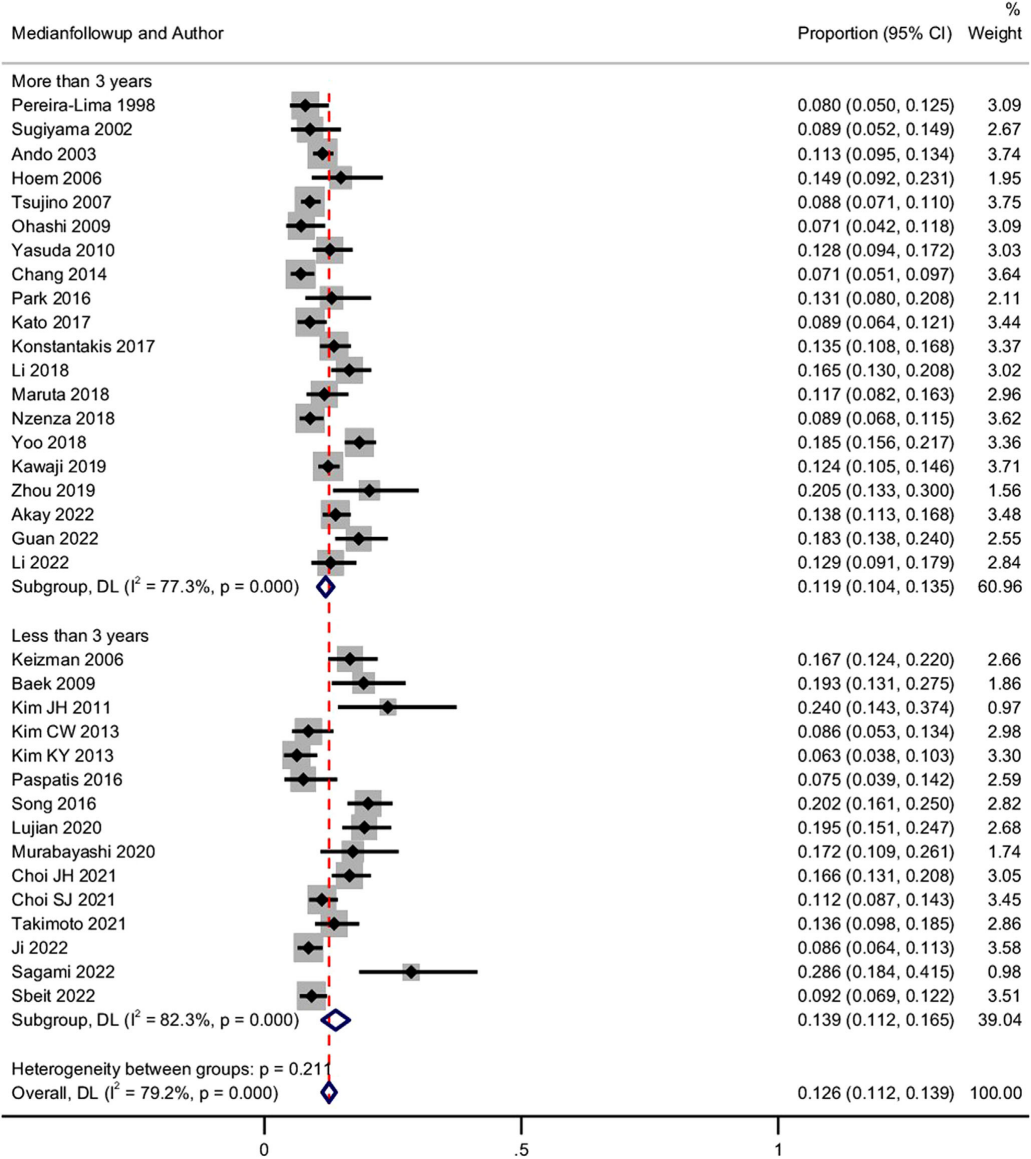
Forest plot for the pooled incidence of recurrence bile duct stone after initial endoscopic clearance with subgroup analysis based on median duration of follow‐up. *Note*: Weights and between‐subgroup heterogeneity test are from random‐effects model

**FIGURE 3:**
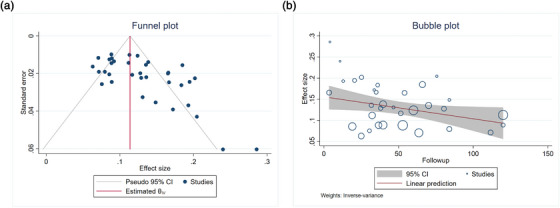
(a) Assessment of publication bias with funnel plot and (b) Bubble plot for meta‐regression for the effect of duration of follow‐up on heterogeneity in the recurrence of bile duct stone.

Overall, 35 studies (*n* = 11,771) reported the recurrence of CBD stones after initial clearance with ERCP.^1^, ^2^, ^3^, ^4^, ^5^, ^6^, ^11^, ^12^, ^13^, ^14^, ^15^, ^16^, ^17^, ^18^, ^19^, ^20^, ^21^, ^22^, ^23^, ^24^, ^25^, ^26^, ^27^, ^29^, ^30^, ^31^, ^32^, ^33^, ^34^, ^35^, ^36^, ^37^, ^38^, ^39^, ^40^, ^41^ The pooled event rate for the recurrence of CBD stones was 12.6% (95% confidence interval [CI]: 11.2–13.9; I^2^ = 79.2%). There was no difference in the event rates on subgroup analysis based on the median follow‐up duration (Less than vs. more than 3 years; Figure 2) or the study design (11.2%, 95% CI: 8.9–13.6 with prospective studies vs. 13.0%, 95% CI: 11.3–14.7 with retrospective studies; *p* = 0.445). The funnel plot and Egger's test showed evidence of publication bias (*p* = 0.0000; Figure 3a). On meta‐regression, the duration of follow‐up was a significant contributor to heterogeneity (*p* = 0.0405; Figure 3b), but not sample size (*p* = 0.2135), the proportion of patients with prior cholecystectomy (*p* = 0.1127), or proportion of patients with periampullary diverticulum (*p* = 0.5987).

### Risk factors for recurrence of BDS after initial clearance

**FIGURE 4:**
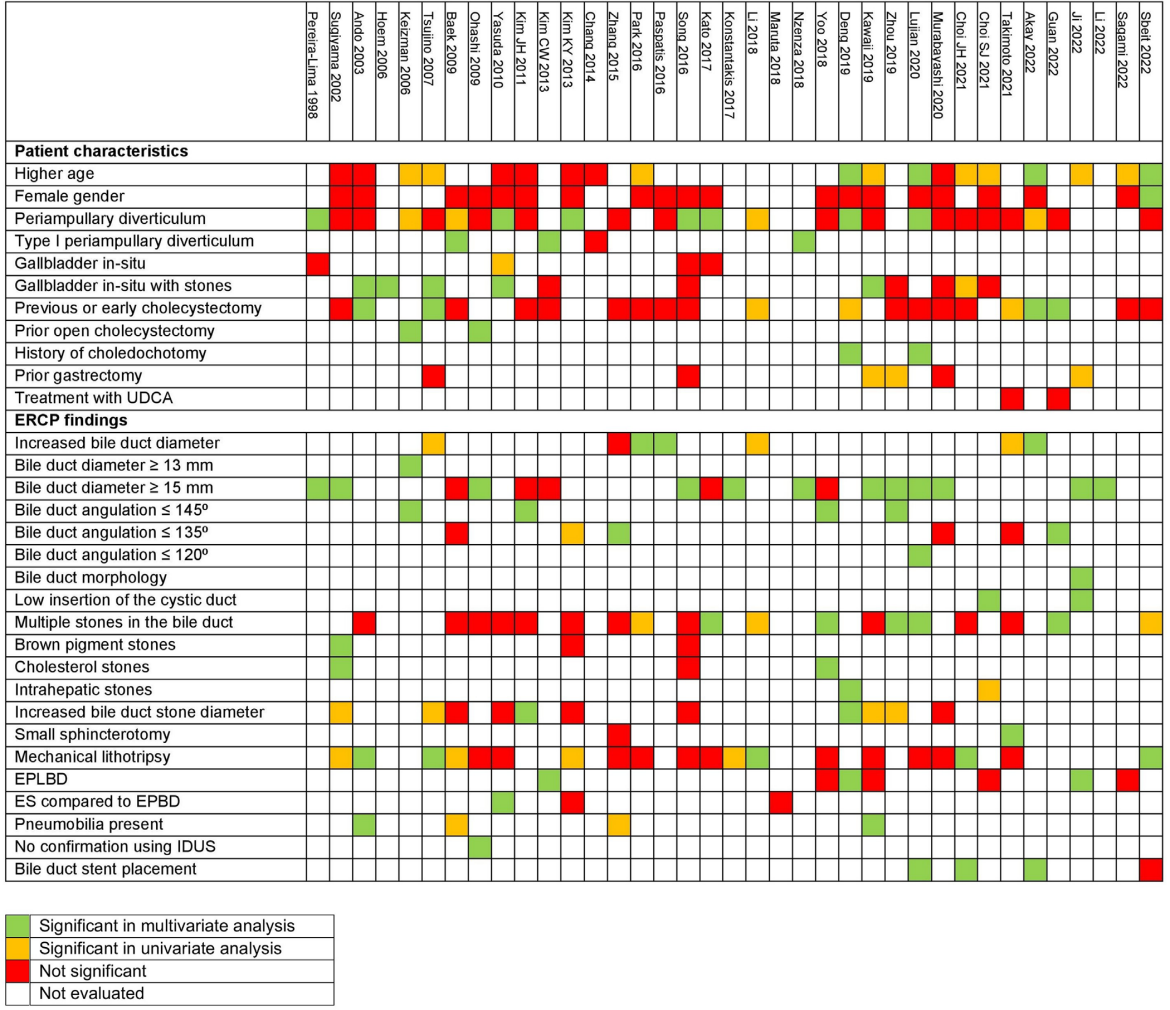
Summary of the predictors of bile duct stone recurrence analyzed in the included studies.

A total of 31 parameters were evaluated in various studies for association with the risk of CBD stone recurrence after initial CBD clearance (Figure 4). A meta‐analysis of outcomes could not be performed as different studies used a different measure of association between exposure and outcome (odds ratio and relative risk).

The most important risk factor was a CBD diameter ≥ 15 mm, which had a significant association with recurrence in 12 studies.^1^, ^2^, ^13^, ^21^, ^23^, ^25^, ^29^, ^30^, ^31^, ^32^, ^38^, ^39^ Other risk factors with significant association with recurrence in three or more studies were reduced angulation of the bile duct (seven studies),^5^, ^14^, ^17^, ^27^, ^30^, ^31^, ^37^ the presence of periampullary diverticulum (seven studies),^1^, ^14^, ^16^, ^21^, ^22^, ^28^, ^31^ cholecystectomy (six studies),^3^, ^5^, ^11^, ^13^, ^36^, ^37^ multiple stones in the bile duct (five studies),^22^, ^27^, ^30^, ^31^, ^37^ use of mechanical lithotripsy (five studies),^3^, ^11^, ^24^, ^33^, ^41^ higher age (four studies),^28^, ^31^, ^36^, ^41^ type I periampullary diverticulum (three studies),^6^, ^13^, ^26^ in‐situ gallbladder with stones (five studies),^3^, ^4^, ^12^, ^17^, ^29^ and bile duct stent placement (three studies).^31^, ^33^, ^36^

## DISCUSSION

CBD stones may recur even after complete removal. This may lead to patient dissatisfaction and a high healthcare cost burden. The present meta‐analysis reports a pooled event rate for the recurrence of CBD stones after confirmed endoscopic clearance is 13.0%. The data with respect to recurrent CBD stones is disparate. Lujian et al., in their study of 262 patients, reported a recurrence rate of 19.48% with an average recurrence time of 20.51 ± 9.65 months.^31^ Kim et al. reported a recurrence rate of 5.8%–6.9% with an average follow‐up period of 59 months.^6^ This disparity may reflect selection bias and variable periods of follow‐up among studies. In a study by Konstatakis et al., of the 67 patients who had a recurrence of BDS, 22 (32.83% of the recurrent) had a second recurrence after 35.2 ± 23.2 months, while a third recurrence occurred to six (8.9% of the recurrent) patients at 16.83 ± 15.3 months.^23^ Subgroup analysis in the present meta‐analysis based on the median duration of follow‐up did not show any difference in recurrence rates. However, on meta‐regression analysis, the duration of follow‐up was a significant contributor to the heterogeneity with a negative association with recurrence, that is, with the increasing follow‐up duration, the incidence of recurrent CBD stones was decreased.

### Diameter of CBD

The present meta‐analysis suggests the most important risk factor for recurrent CBD stones is the bile duct diameter. This has been the finding of multiple previous studies. Studies have suggested that with CBD diameter ≥ 15 mm compared with ≤10 mm, the recurrence rate after CBD stone extraction was 19.5% and 4.9%, respectively.^1^, ^20^ In a study by Luijan et al., a CBD diameter ≥15 mm was found to be a high‐risk factor for CBD stone recurrence.^31^ Another study by Konstantakis et al. showed a cut‐off value of ≥13 mm to be associated with a high risk of recurrence of CBD stones, although the cut‐off value did not reach statistical significance, probably due to the small sample size.^23^ The possible hypothesis for this finding may be that motility and drainage may be affected in a dilated bile duct which results in stasis with increased lithogenicity. Also, secondary bacterial infections are more common in this scenario, which may provide a nidus for subsequent stone formation. However, these theories are yet to be validated with large‐scale studies.

### CBD angulation

The angulation of the bile duct was also an independent risk factor for the recurrence of CBD stones. The angulation along the course of the CBD may promote endobiliary stasis and thus predispose to stone formation. The study by Yoo et al. of 894 patients demonstrated that multiple CBD stones, cholesterol stones, and sharp angulation of the CBD (<145 degrees) were independent risk factors for stone recurrence.^26^ Keizman et al. found that the angle of the end bile duct (<135°) was an independent risk factor for stone recurrence after ERCP.^5^ Another study by Lujian et al. found that acute angulation of CBD (<120°) was associated with a higher risk of recurrence.^31^ An acute angulation prevents optimal clearance of the bile duct with an increased risk of stasis.

### Periampullary diverticulum

Other minor risk factors are the presence of PAD, acute CBD angulation, and multiple CBD stones. Although PAD per se is asymptomatic, multiple studies have pointed to its association with clinical conditions like choledocholithiasis and pancreatic disorders.^23^, ^42^, ^43^ PAD can be classified according to size as well as location. Pereira‐Lima et al. reviewed 203 post‐endoscopic sphincterotomy patients and concluded that the presence of PAD is a strong predictor of bile duct stone recurrence after endoscopic stone extraction similar to bile duct dilatation larger than 15 mm.^1^ A study by Kim et al. focused on the effects of PAD type and size on the clinical characteristics of patients with CDL. They found that, whereas the size of PAD was related to the diameter of CBD, the risk of recurrence was related to the type of PAD. Recurrent CBD stones were more common with PAD type 1 as compared to other types.^15^ Also, PAD was associated with larger CBD stones and greater severity of cholangitis in these patients. Sugiyama et al. proposed the reason for increased recurrence in these patients was due to bile reflux from PAD.^2^ Also, the mechanical pressure on the distal CBD and its proximity to the major duodenal papilla possibly hinder normal bile flow and influence stone formation.^2^

### Multiple BDS at baseline

The present meta‐analysis shows the recurrence rate of CBD stones is higher with the presence of multiple CBD stones at index ERCP. Prior studies have shown that the presence of multiple CBD stones (≥2) is a risk factor for the recurrence of CBD stones after ERCP.^5^, ^12^ Lujian et al. also found that the presence of multiple CBD stones was an independent risk factor for stone recurrence.^31^ A study by Kato et al., in 384 patients, demonstrated that the presence of multiple stones in six of their cohort of 20 patients with stone recurrence was an independent risk factor (RR = 2.44, 95% CI: 1.09–5.44).^22^ The proposed reason is that multiple stones and multiple procedures required to remove them may lead to irreversible damage to the ampullary sphincter. This results in a drop in pressure in the CBD, resulting in the reflux of intestinal contents and bacteria which eventually leads to the recurrence of stones.

### Status of gallbladder

In patients with stones in both the bile duct and gallbladder, cholecystectomy is generally recommended after endoscopic sphincterotomy and clearance of BDS. However, the role of cholecystectomy in patients with BDS without gallbladder stones is controversial. Five studies reported that an in‐situ gallbladder with stones increases the risk of CBD stone recurrence,^3^, ^4^, ^12^, ^17^, ^29^ while six studies reported that cholecystectomy increases the recurrence risk.^3^, ^5^, ^11^, ^13^, ^36^, ^37^ The release of the stored bile by the gallbladder in bulk has a flushing action, which prevents stone formation in the bile duct. With cholecystectomy, this function is lost, leading to an increased risk of BDS. Lau et al. reported that cholecystectomy after endoscopic removal of BDS was shown to reduce recurrent biliary events.^44^ However, Park et al. demonstrated that a past history of cholecystectomy increased the likelihood of bile duct stone recurrence after CBD exploration.^19^ They concluded that among Asian populations without stones following ERCP stone removal, preventive cholecystectomy was not necessary. Hence, cholecystectomy may reduce CBD stone recurrence in patients with gallbladder stones. However, the risk of stone recurrence is independent of cholecystectomy in those without gallbladder stones.

### Use of mechanical lithotripsy

Five studies have shown that mechanical lithotripsy was associated with a higher risk of recurrent calculi.^3^, ^11^, ^24^, ^33^, ^41^ Larger stones are typically seen in settings with an increased tendency for intraductal calculi formation. Hence, the internal milieu may lead to increased calculi formation despite clearance during index ERCP. The other possible explanation is that there may be retained residual fragments of the stones after lithotripsy. These fragments act as niduses, which nucleate over time and increase in size, leading to recurrent CBD stones.

### Bile duct stent placement

The presence of a stent for a prolonged duration in the CBD has been shown to increase the risk of CBD stone formation.^31^, ^33^, ^36^ An in‐dwelling bile duct stent may alter bile duct dynamics, increasing the risk of cholestasis. The median duration of patency for a bile duct stent varied from 2–3 months. Adhesion and accumulation of the bile salt inside the stent lead to stent blockage with resultant sludge and stone formation. Also, the CBD's mucosa may become inflamed due to an increased bile concentration, precipitating inflammatory cells and resulting in stone recurrence.^36^ Hence, in the absence of gallbladder stones and in patients who have undergone cholecystectomy, the bile duct stent should be removed as early as possible.

To the best of our knowledge, this is the first systematic review that looks comprehensively at the risk of CBD stone recurrence and its risk factors. Contemporary studies with relatively large sample sizes were included. Sub‐group analysis based on a median duration of follow‐up was done in order to overcome the variation in follow‐up periods among the chosen studies. Despite this, there are multiple limitations warranting discussion. First, most of the studies included were retrospective in design with inherent limitations of patient selection bias, incomplete information, and the presence of clinical or endoscopic confounders. Secondly, information on stone characteristics like size, morphology, and composition was not available in all of the included studies, which may have clinical significance for stone recurrence. Thirdly, most of the studies were undertaken in large tertiary care centers specializing in advanced endoscopic procedures, which may have led to referral bias. Fourth, we could perform a meta‐analysis for each risk factor due to variations in the use of the relative effect (odds ratio and relative risk). Fifth, the effect of bile microbiome on the recurrence of CBD stone could not be assessed. Lastly, the assessment of complete clearance of CBD at index ERCP with EUS was not assessed. A recent study by Sagami et al. showed that the substantial incidence of recurrent CBD stones can be reduced with the use of EUS. The novel two‐step check method was able to detect 35.7% of the patients who had undergone prior endoscopic clearance and/or cholecystectomy and were subsequently removed via ERCP.^40^

In conclusion, the recurrence of CBD stones after a supposed endoscopic clearance is substantial. A multitude of risk factors are associated with increased risk. Increased bile duct diameter, presence of periampullary diverticulum, reduced angulation of the bile duct, and presence of multiple stones in the bile duct have associations in a higher number of studies. Baseline assessment of these risk factors at index ERCP may help keep a close follow‐up in patients at high risk for recurrent CBD stones.

## CONFLICT OF INTEREST STATEMENT

None.

## ETHICS STATEMENT

Not applicable for systematic review.

## Data Availability

Not applicable as no new data were generated or analyzed in this study.
